# Ab Interno Intraluminal Stent Insertion for Prolonged Hypotony After PreserFlo MicroShunt Implantation

**DOI:** 10.7759/cureus.60221

**Published:** 2024-05-13

**Authors:** Yusaku Miura, Ken Fukuda, Kenji Yamashiro

**Affiliations:** 1 Ophthalmology, Kochi University, Nankoku, JPN

**Keywords:** glaucoma, ab interno, intraluminal stent, hypotony, preserflo microshunt

## Abstract

This study aimed to present an effective and minimally invasive method for treating prolonged hypotony after PreserFlo MicroShunt (PMS) implantation, which can cause serious complications. A 79-year-old man with primary open-angle glaucoma of the right eye underwent ab interno intraluminal stent insertion for prolonged hypotony after PMS implantation. After making two corneal incisions at the 5 and 8 o'clock positions in the right eye, a viscoelastic material was injected into the anterior chamber. A 10-0 nylon suture was inserted into the anterior chamber through a corneal incision in the 5 o'clock position. Next, the 10-0 nylon suture was grasped and inserted into the PMS lumen as a stent with forceps, following which it was cut approximately 1 mm from the tip of the PMS using micro-iris scissors. Finally, the viscoelastic material in the anterior chamber was washed with a balanced salt solution, and self-closure of the two corneal incisions was confirmed. After ab interno intraluminal stent insertion, hypotony improved and stabilized at approximately 10 mmHg. The shallow anterior chamber, choroidal detachment, and hypotonic maculopathy improved rapidly. This novel technique demonstrated effectiveness and minimal invasiveness in treating prolonged hypotony after PMS implantation.

## Introduction

PreserFlo MicroShunt (PMS) (Santen Pharmaceutical Co., Ltd., Osaka, Japan) implantation is an ab externo drainage shunt procedure used to drain aqueous humor from the anterior chamber into the sub-Tenon/subconjunctival space [1]. A recent study found that PMS was less effective in lowering intraocular pressure (IOP) compared to trabeculectomy (TLE) [2]. However, PMS implantation is less invasive than TLE because it does not require scleral flap creation or iridectomy and requires fewer postoperative interventions [2]. Consequently, in the future, it may be frequently utilized as the first-choice filtration surgery remedy. A PMS shunt is 8.5 mm long and has a lumen of 70 μm. Owing to its design and dimensions, this device should theoretically maintain flow at a rate that sustains an IOP greater than 5 mmHg, according to the Hagen-Poiseuille law of pressure gradients [1]. In practice, however, the most frequent postoperative complication of PMS implantation is hypotony, which occurs in 11.1-39% of the eyes [2-7]. Although most cases of postoperative hypotony resolve spontaneously, other secondary complications have been reported, including shallow anterior chambers, hypotony maculopathy, choroidal detachment, and choroidal hemorrhage [4-7]. Prolonged hypotony may require anterior chamber injections of a balanced salt solution (BSS) or viscoelastic material, which may not be sufficient to elevate the IOP. Reportedly, hypotony was improved by incising the conjunctiva to expose the posterior edge of the PMS and inserting a 10-0 nylon monofilament suture into the PMS lumen [8]. However, this method requires conjunctival incisions and sutures. Here, we report a case in which a 10-0 nylon suture was inserted ab interno into the PMS lumen to improve prolonged hypotony after PMS implantation.

## Case presentation

We report the case of a 79-year-old man with primary open-angle glaucoma of the right eye. He had previously undergone cataract extraction, intraocular lens insertion, and trabeculotomy of the right eye. The preoperative best-corrected visual acuity was 0.15 LogMAR in the right eye and 1.0 in the left eye. Goldmann applanation tonometry revealed that the IOP was 16 mmHg in both eyes treated with four glaucoma medications (carteolol/latanoprost fixed combination and brimonidine/brinzolamide fixed combination). The mean deviation on Humphrey visual field 24-2 SITA Standard was -6.78 dB in the right eye and -24.83 dB in the left eye. Slit-lamp examination revealed normal superior conjunctiva. The IOP reduction in the right eye was inadequate, and glaucoma medications failed to inhibit the progression of glaucoma. Therefore, the patient underwent PMS implantation to reduce the IOP in the right eye.

PMS implantation was performed in accordance with previously published recommendations [4]. Briefly, after performing a limbal conjunctival and Tenon’s peritomy between 10 and 12 o’clock in peribulbar anesthesia using a sub-Tenon injection of 2% lidocaine, mitomycin C 0.4 mg/mL soaked sponges were applied under the sub-Tenon space for 5 minutes and then thoroughly washed with BSS. A double-step knife was used to create a scleral tunnel at the 11 o’clock position 3 mm from the limbus. The PMS was then inserted into the scleral tunnel, and the wings of the device were secured to the tunnel. After confirming aqueous humor outflow from the distal end of the PMS, the tenon sac and conjunctiva were fixed to the sclera near the corneal ring using an 8-0 vicryl suture.

**Figure 1:**
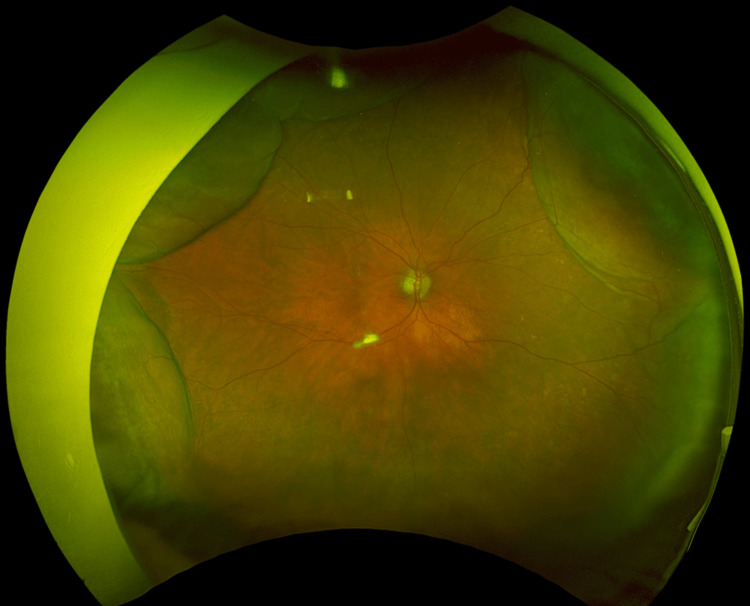
Choroidal detachment by wide-field fundus photography.

No intraoperative complications were observed. The patient was treated postoperatively with 0.5% moxifloxacin four times daily, 0.1% betamethasone sodium phosphate four times daily, and 0.1% bromfenac sodium hydrate twice daily. On postoperative day 1, the right IOP was 3 mmHg, the bleb was tall and diffuse, and the anterior chamber was deep. On postoperative day 2, the right IOP was 3 mmHg; a shallow anterior chamber, choroidal detachment in two quadrants (Figure 1), and hypotony maculopathy were observed; and corrected visual acuity decreased to 0.2 LogMAR. On the same day, viscoelastic material (1% sodium hyaluronate; Opegan Hi, Santen Pharmaceutical, Osaka, Japan) was injected into the anterior chamber. On postoperative day 3, the right IOP was 10 mmHg, the anterior chamber deepened, and choroidal detachment disappeared. However, the injection of viscoelastic material into the anterior chamber had only a temporary effect, and a shallow anterior chamber, choroidal detachment, and hypotony maculopathy recurred a few days after injection. The viscoelastic material was repeatedly injected into the anterior chamber on postoperative days 4, 7, 9, 12, and 17. Considering the need to limit the filtration volume, surgical treatment was deemed necessary, and a second surgery was performed on postoperative day 21.

**Figure 2:**
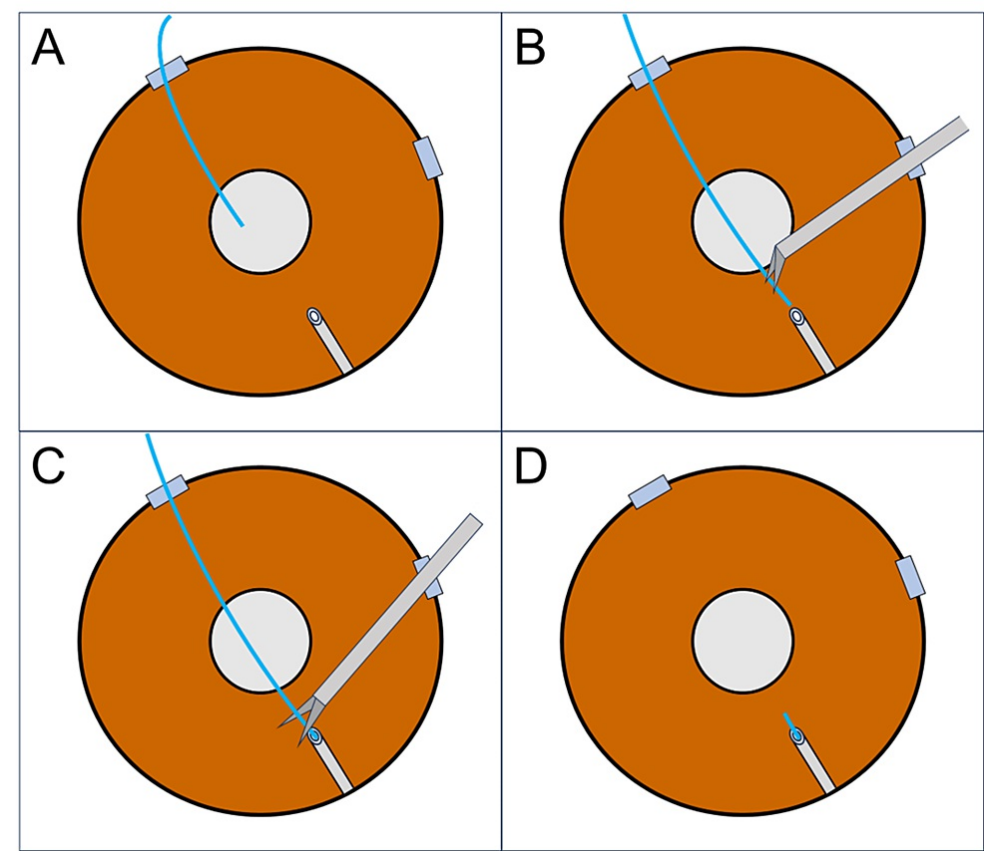
Schematic illustration of ab interno intraluminal stent insertion from the surgeon’s view. (A) A 10-0 nylon suture was inserted into the anterior chamber after injecting the viscoelastic material into the anterior chamber. (B) The 10-0 nylon suture was inserted into the PMS lumen using forceps. (C, D) The 10-0 nylon suture was cut with iris scissors approximately 1 mm from the PMS tip.

Approximately 0.6 mm corneal incisions were created at the 5 and 8 o’clock positions using a 20-gauge microvitreoretinal knife (Mani, Utsunomiya, Japan) under peribulbar anesthesia using a sub-Tenon injection of 2% lidocaine. Viscoelastic material was injected into the anterior chamber through a corneal incision at the 8 o’clock position. At the 5 o’clock position, a 10-0 nylon suture (MANI Suture Nylon; MANI, Japan), with the needle removed, was inserted into the anterior chamber (Figure 2A). The 10-0 nylon suture was grasped and inserted into the PMS lumen with micro anterior lens capsule forceps inserted through the corneal incision at the 8 o’clock position (Figure 2B). After inserting the 10-0 nylon suture as far as possible, the 10-0 nylon suture was cut using micro-iris scissors inserted through the 8 o’clock corneal incision approximately 1 mm from the tip of the PMS (Figure 2C, 2D). The BSS was injected to flush out the viscoelastic material in the anterior chamber, and the procedure was completed after confirming a self-closure of the two corneal incisions.

**Figure 3:**
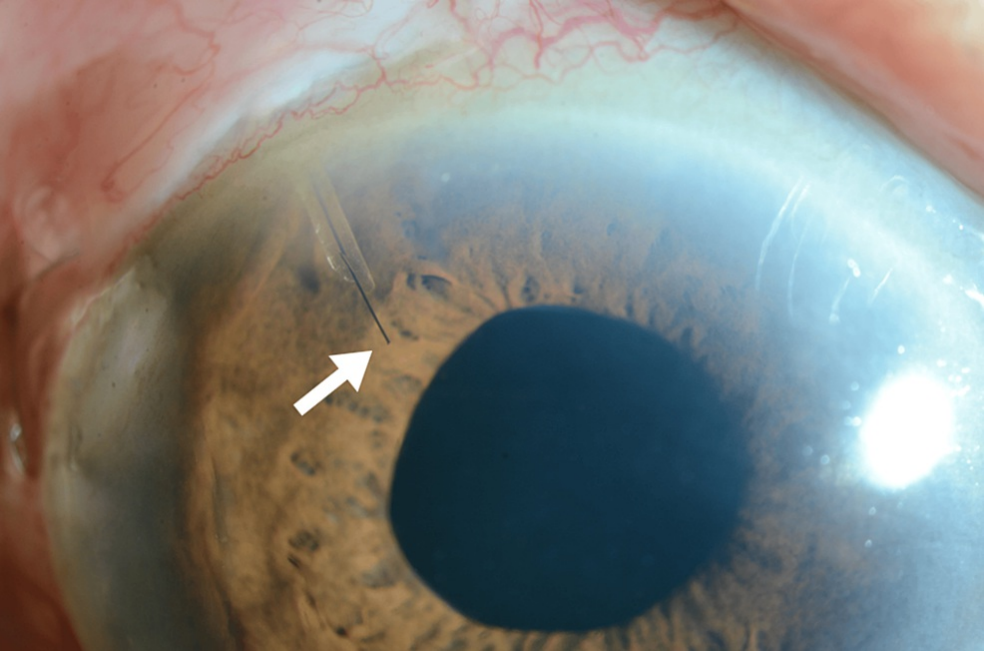
A 10-0 nylon suture inserted into the tip of PMS lumen by slit-lamp (arrow) PMS.

**Figure 4:**
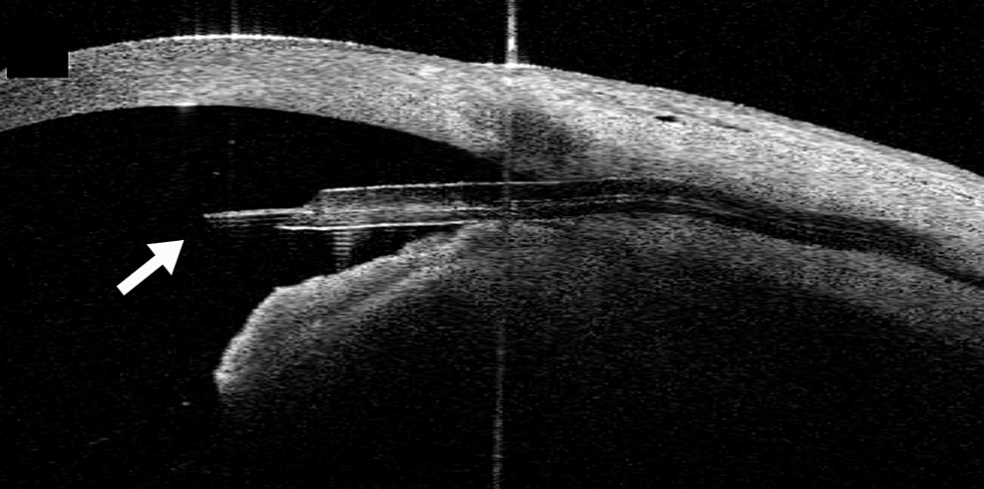
A 10-0 nylon suture inserted into the tip of PMS lumen by anterior segment optical coherence tomography (arrow).

One day after the second surgery, the IOP increased to 6 mmHg, and the anterior chamber was deeper. Choroidal detachment gradually improved. Two months after the second surgery, the right IOP was 13 mmHg, and a brimonidine/brinzolamide fixed combination was started. Four months after the second surgery, the corrected visual acuity was 0.22 LogMAR. The right IOP was maintained at 10 mmHg, and there was no shallow anterior chamber, choroidal detachment, or hypotonic maculopathy. There was no dropout of the 10-0 nylon suture from the tip of the PMS (Figures 3, 4).

## Discussion

Postoperative hypotony after PMS implantation can be caused by overfiltration from the PMS lumen, overfiltration from the scleral tunnel due to poor fixation of the PMS fin, or suppression of aqueous humor production due to postoperative inflammation. The latter two causes are likely to improve spontaneously and normalize IOP. In our case, the fin was securely fixed in the scleral tunnel, and no filtration was confirmed intraoperatively. In addition, a tall and diffuse filtration bleb that developed after surgery suggests that aqueous humor generation was not reduced. Therefore, in this case, hypotony was considered to be due to overfiltration from the PMS lumen.

Prolonged hypotony requires the formation of a deep anterior chamber as soon as possible because the anterior chamber becomes shallow, and the tip of the PMS may contact the cornea, resulting in corneal endothelial cell damage. Choroidal hemorrhage and hypotonic maculopathy may result in serious vision loss. However, there have been few reports on the treatment of prolonged hypotony after PMS implantation. Injection of BSS or viscoelastic material can increase IOP and deepen the anterior chamber; however, they only have a temporary effect. In our case, repeated injections of the viscoelastic material did not improve the hypotony caused by overfiltration from the PMS lumen. Therefore, the fundamental solution may be to increase the resistance to aqueous humor outflow and reduce the filtration volume by inserting nylon sutures into the PMS lumen.

Lupardi et al. reported an insertion of a 10-0 nylon monofilament suture through the distal end of the PMS by incising the conjunctiva [8]. In this study, an increase in the IOP was observed immediately after the insertion of a nylon suture as a stent, which is considered an effective method. However, this method is an ab externo approach that requires incision of the conjunctiva and tenon, which may be highly invasive. Redissection of the conjunctiva and tenon may also increase the risk of leakage from the incision and exposure of the conjunctiva to the PMS. Surgical invasion may also adversely affect the efficiency of absorption of the filtered aqueous humor from the distal end of the PMS by the inner wall of the filtration bleb. By contrast, our method is an ab interno approach that does not require conjunctival or Tenon incisions. Only two small corneal incisions were made, which may have been less invasive and more convenient. Moreover, since the 10-0 nylon suture protrudes from the tip of the PMS in the anterior chamber, it can be easy to remove the 10-0 nylon suture by ab interno approach if the IOP increases after this procedure.

The limitation of this technique is that it is unclear before inserting which thickness of the nylon suture, including a 10-0, 9-0, or 8-0 nylon suture, is the most effective in stabilizing IOP. In this case, IOP stabilized with the insertion of a 10-0 nylon suture. However, if the hypotony does not improve with a 10-0 nylon suture, the insertion of an 8-0 or 9-0 nylon suture should be considered. Moreover, the distance between the PMS tip and the insertion of the nylon suture as a stent to achieve optimal IOP is unknown.

## Conclusions

We described a case of ab interno intraluminal stent insertion using a 10-0 nylon suture that has not been reported before. This technique does not require conjunctival or Tenon incisions. In addition, it reduces aqueous humor filtration volume, leading to increased IOP. Therefore, this technique may be an effective approach for improving prolonged hypotony after PMS implantation.
